# Integrated Water, Nutrient, and Pesticide Management of Huanglongbing-Affected Sweet Oranges on Florida Sandy Soils—A Review

**DOI:** 10.3390/plants11141850

**Published:** 2022-07-14

**Authors:** Qudus O. Uthman, Alisheikh A. Atta, Davie M. Kadyampakeni, Jawwad A. Qureshi, Kelly T. Morgan, Peter Nkedi-Kizza

**Affiliations:** 1 Soil and Water Sciences Department, University of Florida, 2181 McCarty Hall, Gainesville, FL 32611, USA; q.uthman@ufl.edu (Q.O.U.); conserv@ufl.edu (K.T.M.); kizza@ufl.edu (P.N.-K.); 2 Citrus Research and Education Center, University of Florida, 700 Experiment Station Rd, Lake Alfred, FL 33850, USA; 3 Southwest Florida Research and Education Center, University of Florida, 2685 SR 29 N, Immokalee, FL 34142, USA; aatta@ufl.edu (A.A.A.); jawwadq@ufl.edu (J.A.Q.)

**Keywords:** imidacloprid, management, nutrients, pesticide

## Abstract

Citrus greening (huanglonbing (HLB)) disease has drastically reduced citrus fruit production in Florida over the last two decades. Scientists have developed sustainable nutrient management practices to live with the disease and continue fruit production. Best pesticide management practices have been devised to reduce the spread of HLB by Asian citrus psyllid (ACP). However, soil application of excessive nutrients and the use of soil drench application of pesticides to huanglongbing-infected citrus groves have been a serious environmental concern since the recent development of resistance to some pesticides. It is important to understand the consequences of applying pesticides and nutrients beyond the recommended application rates with an inappropriate method for citrus growth and development. Alternatively, foliar sprays of some nutrients proved effective to meet plants’ requirements, and foliar sprays of effective insecticide products could provide an adequate mode of action for group rotation to minimize insecticide resistance by ACP and other pests. Sustainability in citrus production systems should include best management practices that improve pesticide and nutrient efficiency by including the total maximum daily load exiting the grove to reduce pesticide and nutrient exports into waterbodies.

## 1. Nutrient Management of HLB-Infected Sweet Oranges Trees

### 1.1. Macronutrients

**Nitrogen:** In Florida, where citrus is the dominant crop, sandy soils, a shallow water table, low soil organic matter, and intense rainfall result in nitrogen (N) leaching beyond the root zone [1,2,3]. When N is deficient in the soil, citrus tree growth is limited, and leaves become chlorotic [4]. N application below the recommended rate for bearing trees for an extended period causes trees to relocate leaf N from the oldest leaves into the new ones and the former will drop prematurely, which leads to a thin plant canopy [5,6,7]. The relative increase in citrus N bioaccumulation is proportional to the relative increase in size over time since it is the most abundant macronutrient in plants [8,9]. Tree canopy volume, leaf area index, fine root density, and root life span have been reported to increase in response to increased N fertilizer and either foliar or ground-applied fertilizers containing Ca, Mg, Mn, and Zn in HLB-infected trees [8,10]. Best management practices (BMPs) for mobile nutrients such as N include split application rates for HLB-infected citrus trees with severe root density deterioration [10]. Combining N fertilizer application with smart irrigation scheduling could reduce soil available N to less than 4.0 mg kg^-1^, which is 2.0–4.0 less than conventional N applications [2,11,12]. Once a citrus tree attains optimum size, further canopy increases are not required to maximize N use efficiency. Thus, N fertilizer management should be emphasized to sustain adequate vegetative growth for the development of branches and restock N crop removal from the soil [8,12]. The citrus industry in Florida should develop sustainable citrus production systems that improve N efficiency by including the total maximum daily load exiting the grove to reduce the input of nutrients exported to water bodies [12,13].

**Phosphorus:** Phosphorus (P) fertilizer is applied based on a soil test index, thus citrus trees receive P at lower rates as compared to N, or may receive nothing if soil test P is high [12,14]. Bearing citrus trees do not respond to P fertilizer except when trees are planted on soils with excess P fixation or soils lacking in P [5]. Citrus responses to P fertilization include improved yield, juice content, soluble solids, acid ratio, and decreased rind thickness [5,15]. Phosphorus is involved in energy transfer within the plant, photosynthesis, and carbohydrates and biomass accumulation is affected by the irrigation method and type of fertilizer used in a citrus grove. Kadyampakeni et al. [16] reported that the amount of P accumulated on HLB-infected citrus trees was highest in drip, followed by restricted micro-sprinkler and conventional irrigation methods. The added fertilizer in response to P biomass accumulation was highest in roots, followed by leaves and fruits [6,17]. Excessive application of P on sandy soils beyond the recommended rate in HLB-infected citrus groves could result in P leaching and runoff, which eventually plays a vital role in the eutrophication of surface water bodies [18]. However, in a split application study of essential nutrients conducted at Immokalee, FL during the 2017–2019 growing seasons, leaf P was within the optimum range without the addition of any P to the soil during any season of the study [19].

**Potassium:** Nitrogen contributes the greatest impact on vegetative tree growth, flowering, and fruit yield, while potassium (K) plays a crucial role in determining yield, fruit size, and quality [16]. Low yield, small fruit, fruit splitting, and/or wrinkled fruit skin are symptoms of K deficiency [20,21]. If trees fail to respond to soil K application, foliar application of potassium nitrate (KNO3) or monopotassium phosphate (KH2PO4) can be an alternative approach to enhance leaf K as the inherent characteristics of soil fixed K [5]. Thus, recurrent K fertilizer application is necessary in citrus groves. Potassium is important for phloem functioning; hence, changes in its concentration within the plant can have a remarkable influence on phloem physiology [22]. High sugar accumulation in the leaves of K-deficient plants does not stimulate an increase in root sugar content or growth [23]. Potassium applied for optimum plant growth normally suppresses the vulnerability of plants to pathogens [24,25]. Since K plays a vital role in the development of cell walls, K deficiency results in reduced synthesis of proteins, starch, and cellulose that enable the pathogen to easily penetrate plant tissues. This implies that roots deficient in K are more vulnerable to infection by pathogens either in the soil rhizosphere or from other diseased organs [25,26]. Pathogens themselves utilize nutrients, which decreases availability to the plant, and thereby increases its vulnerability to secondary contagions due to nutrient deficiency [25,27,28,29].

**Calcium:** Calcium (Ca) is involved in cell wall development and contributes to the structural integrity of plant membranes such that Ca deficient plants are more vulnerable to fungal pathogens [26,27,30]. Thus, Ca has been known to be involved in plant disease resistance, and Ca-deficiency may cause leakage of sugars and amino acids from symplast to apoplast, which then serves as a food source for pathogens [31]. Previous studies indicated that HLB-infected leaves were significantly lower in Ca concentration than HLB-free citrus leaves [26,27,32,33]. The apparent decrease in Ca for HLB-infected citrus trees could be attributed mainly to limitations in nutrient uptake and transport [34] and could be considered a major feature of HLB-induced physiological disorders [32]. Calcium thiosulfate treated HLB-infected trees showed an increase in fine root density of 11% (<2 mm diameter), 1.4×–5.3× greater median root lifespan [10,35,36], and 3.1×–3.5× more leaf area index compared with control trees [11]. Meanwhile, research results also indicated that Ca-treated trees showed 2× greater irrigation efficiency per unit leaf area as compared with control HLB-affected citrus trees under drip and restricted microsprinkler irrigation methods [11,37].

**Magnesium:** Magnesium (Mg) is an essential element involved in the structure of chlorophyll, cell division, and carbohydrate synthesis [38], photosynthetic carbon dioxide fixation, photo-assimilate phloem loading, and partitioning [23]. Mg enhances the uptake and transport of P, is involved in nucleic acid metabolism, and influences the mobility of carbohydrates from the source (leaves) to the sink [38]. Mg deficiency in the soil is reflected in plants as the translocation of Mg from leaves to the developing fruit and from older leaves to young leaves on the same shoot [23]. Since carbohydrate accumulation is prevalent in Mg deficient leaves, this will induce a reduction in leaf growth, eventually affecting the translocation of sucrose to the roots [20,23]. Foliar Mg concentrations in the leaves of HLB-infected trees were found to be lower than in the leaves of HLB-free citrus trees [26,27,29]. Generally, leaf nutrients were higher in HLB-free citrus trees than in HLB-infected trees in Hamlin oranges [31], and in Siem mandarins surveyed at three locations in Indonesia receiving soil or foliar applied Mg fertilizers on either sandy or clay-loam soils [29]. Magnesium thiosulfate treated HLB-infected Hamlin citrus trees showed an increment of 11–34% more fine root density, 1.1×–3.1× greater median root lifespan [10], and 1.3×–1.5× more leaf area index [11] as compared with control trees. Studies have also demonstrated that the high availability of cations Ca, K, and Mn can lead to large decreases in Mg root uptake [32,39]. However, it has been commonly determined that Mg concentration in roots is not influenced by K-Mg antagonism, but the transport from root to shoot is reduced by high soil K concentration. Since Mg is a mobile element, it may leach into sandy soil with excessive rainfall, which contributes to acidification and poses a threat of insufficient nutrient uptake by citrus trees [40]. Previous research has shown that adequate leaf Mg can reduce large leaf drop and twig dieback, increase fruit production, and improve fruit quality [39,41]. Meanwhile, the leaf Mg concentration was below the optimum range in HLB-infected trees. However, putting Mg as thiosulfate on the ground increased the amount of Mg in the leaves and kept it within the best ranges [19,31,33,42].

### 1.2. Micronutrients

**Manganese:** Manganese (Mn) deficiency symptoms are usually notable on HLB-infected trees and were not deemed deficient in HLB-infected leaves due to high leaf starch accumulation caused by HLB [31,33,42]. Manganese is a crucial micronutrient involved in the formation of photosynthetic proteins and enzymes [43]. Its deficiency in leaves affects the water splitting system of photosystem II, which supplies the required electrons for photosynthesis [43]. Nevertheless, Mn concentrations were lower in HLB-infected plant leaves than in HLB-free leaves [26,32]. Researchers reported up to 80% lower Mn of root tissues and 58% in the leaf tissue concentration of HLB-infected than HLB-free citrus trees [44]. In foliar and ground-applied Mn and Zn studies on HLB-infected citrus, fine root length density (1–2 mm root diameter) showed an increase of 1.8×, 3.5×, and 1.5× greater in the spring and 7.8×, 5.3×, and 1.5× greater in the summer than the untreated trees in response to 9 kg ha^−1^, 18 kg ha^−1^, and 27 kg ha^−1^ metallic Mn and Zn treatments, respectively [10]. Meanwhile, the median root life span of HLB-infected citrus showed 1.1×, 1.25×, and 0.75× greater than the control trees in response to 9 kg ha^−1^, 18 kg ha^−1^, and 27 kg ha^−1^ metallic Mn and Zn treatments, respectively [10]. Previous studies indicated that root elongation and vegetative growth were hampered by excess Mn (2500 µM), treatments in conifer *P. menziesii* var. *glauca* and *P. menziesii* var. *viridis* trees [43]. Similarly, the yield was significantly impacted by the application of MnSO_4_ in which the yield of the 17 kg ha^−1^ Mn/year treatment was 45% greater than that of the unsprayed control; however, the yield was reduced by 25% for the 17 kg ha^−1^/year treatment [42].

**Zinc:** Although zinc (Zn) deficiency symptoms are normally evident on HLB-infected trees, the physiological impact of this nutrient on the leaves and fruits has not been identified yet [45]. Phloem translocation of Zn is one of the important features that contribute to HLB-induced Zn deficiency in grapefruit [38,44]. Research has shown that there is indirect evidence for high Zn translocation in the phloem of HLB-free citrus trees, which had previously been deemed to be an element with intermediary phloem mobility [32,44]. One of the striking features of Zn is that it has a mixed impact on disease severity by increasing in some instances and decreasing in others [27]. The Zn concentration was reported to be higher [26] and lower [33,44] in HLB-infected citrus trees in the field as well as in pot trials. Nevertheless, Zn leaf concentration did not differ between treatments (foliar only or foliar and soil applied fertilizers) or sites (sandy or clay-loam soils) [29,31]. The reason for such mixed results could be due to several factors, including other diseases such as Phytophthora, excess application of fertilizers by growers [26], or molecular physiology involved in nutrient-disease interactions, which are not well understood [27]. Zinc levels in mature grapefruit leaves were higher than in young grapefruit leaves in both HLB-free and HLB-infected plants. Zinc levels in HLB-infected leaves were significantly lower than in HLB-free leaves because the phloem in HLB-infected trees moved less Zn [38,44].

**Boron:** Boron (B) is one of the most vital micronutrients but is less understood compared to Zn [31,46,47]. A decrease in leaf B has been identified in HLB-infected citrus trees [31,46,47]. Boron is involved in carbohydrate transport through the phloem and cell wall structure, and hence influences pathogen susceptibility [31]. Some of the symptoms associated with low B are phloem disintegration, “corky” leaf veins, and “hard fruit”. The latter symptom is developed as the fruit turns firm and dry, caused by the swelling of the fruit peel and by gum impregnations [31,47]. The symptom of B shortage in sink tissues because of inadequate uptake of B from soil suggests that the rate of phloem B translocation is roughly determined by B uptake [48]. Under B deficiency, about 95 to 98% of B is accumulated in cell walls in cultured squash leaves [18] and tobacco cells [18,49]. The mechanism of root uptake and subsequent B transport is not well understood [50]. However, passive diffusion is considered the mechanism of B root uptake in higher plants [50,51]. Boron tends to accumulate in the margins of older leaves because B moves along the transpiration stream and accumulates at the end of the transpiration stream [51]. Soil B decreased with Ca application to the soil [11], yet no significant effect was found with B application on leaf B concentration, and it was within the optimum ranges at the Lake Alfred, FL and Immokalee, FL sites [19,52].

## 2. Asian Citrus Psyllid and Its Pathogen

Asian citrus psyllids (ACP) (Diaphorina citri) are prolific, and females can lay eggs a day after mating. Under ideal conditions (25 °C) and relative humidity (60–80%), they can lay 600–700 eggs on average [53]. ACP has five nymphal instars stages before developing into adults and the females live longer than their males [54]. ACP flight is prompted by daylight and pronounced during warm, sunny afternoon hours [55]. Their flight is affected by changes in barometric pressure and air temperature [56]. High ultraviolet (UV) light at high altitudes is probably the cause of the low ACP population [57]. Young, expanded citrus leaf flush between 1–5 days of age is called “feather flush” and is a preferable host of ACP [58]. ACP transmits the pathogen Candidatus liberibacter asiaticus (CLas) causing huanglongbing (HLB) or greening disease from its adult ACP to nymph ACP via their host (leaf flush). This mode of dispersal is called “flush transmission” [59]. The yellowish color of infected citrus leaves attracts adult ACP, and they subsequently move to healthy parts of the citrus plant due to poor nutrition in the infected primary hosts [60].

## 3. Insecticides for Controlling Asian Citrus Psyllid (ACP)

Insecticides play an important role in ACP management [61]. The systemic neonicotinoid insecticides (imidacloprid, clothianidin, thiamethoxam, and cyantraniliprole) are allowed in Florida citrus to be soil applied with rate restrictions to young trees [61,62,63]. Foliar sprays of insecticides before flushing during tree dormancy have also proved to be effective in reducing ACP populations [61,63]. The prevalence of HLB in Florida citrus groves has greatly escalated the use of insecticides to control its vector (ACP) [64]. However, efficacy, product availability, application equipment availability, pest pressure, conservation of beneficial insects, and resistance management affect the timing, choice of products, and application methods during the growing season aimed at maximizing the benefits while reducing negative consequences [61].

The use of neonicotinoid insecticide is growing rapidly in citrus protection against the piercing and sucking insect vector of CLas [65]. Neonicotinoids dominate with 27% of the insecticide market, nearly as much as the current combination of pyrethroids, organophosphates, and carbamates combined [66]. These insecticides are selective and act on the molecular target site of nicotinic acetylcholine receptors (AChR)—an ion channel (http://www.irac-online.org/, accessed on 1 May 2022). Neonicotinoids bind to a major excitatory neurotransmitter found in the central nervous system called acetycholine on nAChRs to cause hyper-excitation, lethargy, and paralysis [65] (http://www.irac-online.org, accessed on 1 May 2022). Imidacloprid (IMD) is a systemic class of neonicotinoid insecticides for the control of ACP in Florida citrus groves [67]. IMD is a very water-soluble organic molecule that has high mobility in sandy soils and is weakly adsorbed in soils [67,68]. IMD is easily taken up by plant roots and translocated to the entire plant system, which allows piercing and sucking aphids such as ACP to absorb IMD when they feed on the tender leaves [68].

The resistance level to neonicotinoids in ACP nymphs can be higher than in adult ACP. Thus, insecticide rotation objectives can be achieved using the full range of selective insecticides available for any additional sprays [61]. Existing rotation of registered modes of action, as well as incorporation of new modes of action and/or pesticide alternatives, will be important in sustaining the effectiveness of currently available insecticides against ACP [69]. Foliar sprays of effective insecticide products provide an adequate mode of action for group rotation to minimize insecticide resistance by ACP and other pests [61]. However, cyantraniliprole is the available alternative for rotation with soil applied neonicotinoids in Florida. Moreover, foliar sprays of about eight effective insecticides could be rotated over a year without repeating modes of action and with little or no use of neonicotinoids or cyantraniliprole [61].

## 4. Effect of Evapotranspiration (ET) Deficit Irrigation to Retain Nutrients and Pesticides in the Root-Zone of HLB-Infected Citrus Trees

Water management for HLB-infected citrus trees on Florida sandy soils keeps water content at field capacity for the 0–60 cm soil depth (root-zone) to significantly reduce leaching and water evaporation losses [70]. At field capacity water content in the root zone, the matric potential gradient is almost zero, and the main water potential gradient is due to gravity. Thus, the total water potential gradient is approaching unity between the surface soil and the 60 cm depth. Correspondingly, the hydraulic conductivity in sandy soils at field capacity water content is very small, thus by Darcy’s law, the water velocity is essentially numerically equal to the small hydraulic conductivity. Such conditions lead to the retention of water, pesticides, and nutrients in the root zone, with minimal leaching below the root zone. Therefore, supplemental irrigation to meet evapotranspiration demand is sufficient and effective to keep the soil moisture content at or near field capacity [71]. This means that there is sufficient water for nutrient and pesticide uptake by the citrus roots, and the unavailability of any nutrient or pesticide is mainly due to soil adsorption [72].

Using data from Candler fine sand, retardation factors were determined for multiple depths. A retardation factor, by definition, is a measure of the magnitude by which the solute velocity is reduced compared to pore water velocity. A retardation factor of 1 means the solute is not adsorbed and moves with the same velocity as the water. P and K (macronutrients) are strongly adsorbed in the 0–30 cm soil depth and their downward movement is highly retarded at field capacity soil moisture content (Table 1 and Table 2). Mn and Zn (micronutrients) are highly retarded and strongly adsorbed to the soil (Table 1 and Table 2) and were not available to the plants [72,73]. B has a relatively low adsorption coefficient and can be taken up by plants [52]. IMD, with a relatively high sorption coefficient compared to B (Table 1 and Table 2), was also taken up by citrus but was reported to leach below the root-zone only after a heavy rainfall event [67]. Although water management as outlined keeps micro-nutrients in the root-zone, their availability for plant uptake in sandy soil depends on the interaction of the nutrients and soil that can render them unavailable to citrus plants in the root-zone.

## 5. Concluding Summary

Best nutrient management practices are needed to withstand the HLB disease pressure in citrus production systems. Although nutrient management of HLB-affected trees creates the availability of disease hosts that could be responsible for an increase in the ACP population, proper application rates and methods of nutrient application have proven to be effective for the growth and development of HLB-infected citrus trees. However, their deficiencies can be amended by adding these nutrients to the soil, foliage, or a combination of both. While N, Ca, Mg, Mn, and Zn promote vegetative growth, water use and nutrient dynamics, and yield, Ca and Mg specifically increase vegetative, root length density, the life span of fine roots, and fruit yield in HLB-infected citrus trees.

ACP population varies depending on the season and climatic conditions of the environment. Killing the oviposition stage, nymph survival and development stage, as well as adult emergence, can reduce the ACP population [74]. Insecticides such as systemic neonicotinoids (especially imidacloprid) are used in Florida citrus production to control ACP populations. Its resistance can affect the timing and application method during the growing season. While controlling for the ACP, adequate and effective nutrients need to be applied to the HLB-infected trees to sustain the trees for maximum growth and development, or else the tree will die in a short period of time with no fruit production benefits.

## 6. Emerging Research Needs and Questions

Nutrient and pesticide studies have been conducted to improve the management of HLB-infected citrus groves. Combined nutrient and pesticide management have been studied in the field using foliar sprays, but not all the nutrients are best applied as foliar sprays, especially macronutrients. In some cases, there are elevated concentrations of micronutrients (especially Mn and Zn) in the soil that may not be available to the plants, which makes the foliar application method very effective for some micronutrients. Florida citrus growers do mix nutrients and pesticides together in the same tank to avoid multiple applications, but how those nutrients and pesticides interact is yet to be understood. The fate of pesticide and nutrient applications that the Florida growers practice in managing HLB-infected citrus trees has not been evaluated on Florida sandy soils. Thus, understanding the processes involved in the growers’ practices of combined nutrient and pesticide management of HLB-infected citrus trees will enhance better management and sustenance of environmental quality.

## Figures and Tables

**Table 1:** Adsorption coefficients of nutrients and imidacloprid (IMD).

Soil Depth	SOC ^‡^	K_D_					
		P ^†^	K ^†^	B ^‡^	Mn ^‡^	Zn ^‡^	IMD ^Ⴕ^
cm	g g^−1^	mL g^−1^					
0–15	0.003	1.72	1.65	0.10	2.20	6.47	0.64
15–30	0.002	2.05	0.93	0.03	0.13	2.68	0.35

SOC: Soil organic carbon; K_D_: Adsorption coefficient. ^†^ Sourced from [9]. ^‡^ Sourced from [52,72,73]. ^Ⴕ^ Sourced from [67].

**Table 2:** Retardation factor of nutrients and imidacloprid.

Soil Depth	ρ_b_	θ_FC_	R_FC_					
			P ^†^	K ^†^	B ^‡^	Mn ^‡^	Zn ^‡^	IMD ^Ⴕ^
cm	g cm^−3^							
0–15	1.56	0.07	39.33	37.77	3.23	50.03	145.19	15.22
15–30	1.67	0.10	35.24	16.53	1.50	3.17	45.76	6.81

SOC: Soil organic carbon; ρ_b_: Bulk density; θ_FC_: Moisture content at field capacity; R_FC_: Retardation factor at field capacity. ^†^ Sourced from [9]. ^‡^ Sourced from [52,72,73]. ^Ⴕ^ Sourced from [67].

## Data Availability

Not applicable.
